# Can We Do Better Next Time? Italians’ Response to the COVID-19 Emergency through a Heuristics and Biases Lens

**DOI:** 10.3390/bs12020039

**Published:** 2022-02-07

**Authors:** Raffaella Misuraca, Ursina Teuscher, Costanza Scaffidi Abbate, Francesco Ceresia, Michele Roccella, Lucia Parisi, Luigi Vetri, Silvana Miceli

**Affiliations:** 1 Department of Political Science and International Relations (DEMS), University of Palermo, 90134 Palermo, Italy; francesco.cxeresia@unipa.it; 2 Department of Psychology, Portland State University, Portland, OR 97207, USA; ursina@teuscher.ch; 3 Department of Psychology, Educational Science and Human Movement, University of Palermo, 90128 Palermo, Italy; costanza.scaffidi@unipa.it (C.S.A.); michele.roccella@unipa.it (M.R.); lucia.parisi@unipa.it (L.P.); silvana.miceli56@unipa.it (S.M.); 4 OASI, Research Institute-IRCCS, 94018 Troina, Italy; lvetri@oasi.en.it

**Keywords:** heuristics, biases, COVID-19, decision-making

## Abstract

During the outbreak of COVID-19 in Italy, people often failed to adopt behaviors that could have stopped, or at least slowed down, the spread of this deadly disease. We offer cognitive explanations for these decisions, based on some of the most common heuristics and biases that are known to influence human judgment and decision-making, especially under conditions of high uncertainty. Our analysis concludes with the following recommendations: policymakers can and should take advantage of this established science, in order to communicate more effectively and increase the likelihood that people choose responsible actions in a public health crisis.

## 1. Introduction

In January 2020, Italian media and journalists informed the public about the alarming spread of a mysterious lethal virus, of the SARS family, in Wuhan (China). Within only a few weeks of this declaration, the coronavirus disease 2019 (COVID-19) spread outside of China, causing thousands of deaths. The World Health Organization (WHO) declared the COVID-19 outbreak a public health emergency of international concern and a pandemic, on 30 January and 11 March, respectively.

Right after the alarming news broke, during the month of January 2020, the expected response from governors, politicians, and public health experts would have been to react as in any other life-threatening circumstance. Surprisingly, the general response instead was a widespread under-evaluation of the risk; reporters, scientists and experts reassured people that COVID-19 was no more dangerous than a common seasonal flu and that there was nothing to worry about [1,2,3,4,5]. Even in February, when there were already a significant number of victims in Italy (on 29 February there were already 1128 infected people and 29 deaths.), governors’ decisions to lock down the most-hit areas were slow. Information provided to citizens on the actual danger of the disease and the level of control over the spreading of the infection remained extremely unclear and contradictory until the first week of March 2020 [6,7]. In addition, the recommendations about safety measures to adopt, to prevent infection, such as hand washing, face coverings, quarantine, and social distancing [8] were confirmed and disconfirmed several times; wearing a mask, for example, was sometimes considered extremely important and mandatory, other times useless and even dangerous [9,10,11]. This lack of clarity produced a huge degree of uncertainty among Italians and a lack of trust towards the main sources of information. Citizens did not know whom or what to believe.

Research in judgment and decision-making has shown that, to deal with uncertainty, individuals adopt cognitive strategies, or heuristics, in order to simplify the environment [12]. More specifically, heuristics are automatic mental shortcuts, used by humans, to make decisions quickly, when information is complex or incomplete. Sometimes heuristics result in good decisions. Other times, they lead to systematic errors, named *cognitive biases*.

In this manuscript, we first examine the main heuristics and cognitive biases that could explain some of the most common behaviors of Italians during COVID-19 that were problematic from a public health standpoint, such as going to crowded discotheques and bars, refusing to wear a mask and, in general, to respect the safety measures necessary for the control of the spreading of the infection [13,14,15,16]. We then provide solutions to help people engage in more desirable preventive public health behaviors.

## 2. Heuristics and Cognitive Biases during the COVID-19 Pandemic

A key account of how lay people judge risks in uncertain situations is the availability heuristic [12]. It is a mental shortcut that leads individuals to erroneously judge the probability of a given event, based on how easy it is to recall that event from their memory. The easier it is to recall an event, the more that event will be estimated as frequent. This short-cut in our thinking explains why people typically overestimate the frequency of memorable events, even those that are, in reality, extremely rare, such as plane crashes, being killed by a terrorist, or being attacked by a shark [17,18]. Sensational events, such as these, are more often reported in the media, and thus they are easy to recall from our memory. Consequently, people tend to overestimate their probability, and therefore over-react; for example, avoiding travel by plane, to visit a city where there was a terroristic attack in the past, or to avoid swimming in the ocean.

Based on the availability heuristic, when media and politicians announced the risk of the spreading of a deadly viral disease, Italians reacted with an erroneous under-estimation of the danger. Their memory, indeed, is full of episodes in which journalists, and media in general, spread alarmism for no reason. People today know well that the goal of journalists is not merely to provide information, but to gain the greatest number of readers and viewers, which is often achieved by playing to our emotions [19,20]. In other words, Italians are habituated to the fact that the media sensationalize events and, thus, they do not trust them anymore, even when it would be better to believe them.

As media, politicians are no longer trusted by Italians [21]. A recent survey by the research institute Demos [22] showed that 85% of Italians do not trust their own parliament, believing that politicians are mostly driven by personal interest (e.g., to achieve political power or be reelected), rather than by a genuine interest in the general public good [23]. Consequently, citizens have under-evaluated or even completely ignored politicians’ recommendations and policies about the containment of COVID-19.

The availability heuristic is not the only cognitive strategy that can explain the reaction of Italians to COVID-19. Many other heuristics and cognitive biases may have significantly contributed to it. For example, research shows that one way to cope with uncertainty is to imitate others’ behavior [24]. This tendency to believe or do something simply because most people believe it or do it is known as the bandwagon fallacy (also called common belief, consent heuristic, or imitation bias). The psychologist who discovered this phenomenon is Solomon Asch [24]. In his experiment, Asch asked a group of participants to say aloud whether two lines had the same length. In reality, all but one of the participants were actors, who were responding according to precise instructions, provided by the experimenter. Asch observed that the judgment of the non-actor participant was strongly affected by the judgments of the actors, even when their judgments about the length of the lines were clearly wrong. The reasons behind this irrational behavior are twofold, as follows: the preference of individuals to conform to others and the tendency of individuals to derive information from others. The bandwagon effect has been replicated in many different domains [25] and seems to be strongest when individuals are unfamiliar with a certain event or when they have limited possibilities to process the information [26]. Given the high levels of uncertainty that comes with the outbreak of a novel disease, people imitated the behavior of others, such as going outside, gathering with family and friends, or refusing to wear masks. This form of social influence is particularly strong among young people. For example, Steinberg and Monahan [27] showed that younger people (between 10 and 14 years old and between 18 and 30 years old) are less resistant to peer influence. During the COVID-19 outbreak in Italy, young individuals maintained their regular daily routines and social contacts, even when schools and colleges were closed because of the magnitude of the emergency (e.g., hosting house parties, group gathering at parks, travelling, etc.) [28,29].

Another relevant bias is confirmation bias, a general tendency to attribute greater importance to information that confirms, rather than contradicts, our hypotheses. The confirmation bias was first investigated by English psychologist Peter Wason [30], with a reasoning task known as the four-card selection task. In this task, participants were shown four cards, each having a letter on one side and a number on the other side. The cards showed A, D, 4, and 7, respectively. The researcher informed participants that the cards were made according to the following rule: “if a card has the vowel A on one side, it must have the number 4 on the other side”. Participants had to select the two cards that needed to be turned over, in order to determine whether the rule was true or false. The correct solution consists of turning over card A and card 7. The rule can be determined as false if there is a number other than 4 on the back of card A, and if there is the vowel A on the back of card 7. However, Wason observed that the majority of people instead turned over cards A and 4. In other words, rather than check the card that could have falsified their hypothesis, they simply attempted to confirm it, not realizing that such a confirmation did not actually determine whether the rule was true or false [31,32]. Extending this finding to decision-making behavior during the COVID-19 outbreak, people might have erroneously focused their attention more on information that confirmed what they already believed (e.g., that COVID-19 was similar to the common seasonal flu, that journalists disseminate panic and anxiety for personal interest), rather than on new evidence that supported a different conclusion (e.g., the higher transmission rate and number of deaths compared to a common flu).

Related to confirmation bias is the ostrich effect (although we should note that ostriches do not, in fact, bury their heads in the sand to hide from danger: https://www.sciencefocus.com/nature/do-ostriches-really-bury-their-head-in-the-sand/, accessed on 15 November 2021), which refers to the tendency to ignore negative information by “burying one’s head in the sand”. Our natural predisposition to avoid unpleasant situations and psychological discomfort may lead us to ignore problems, rather than coping with the anxiety that comes with them. For example, people sometimes intentionally avoid medical screenings for fear of receiving bad news about their health. The ostrich effect is a very common cognitive bias in financial situations. Investors tend to check, for example, their portfolios more often when markets are going well, rather than when markets are performing poorly [33]. Galai and Sade [34] even found that individuals who were faced with uncertain investments preferred an investment where the risk was unreported, over an investment with a similar risk–return profile for which the risks were frequently reported. As uncertainty increased, so increased the premium that investors were willing to pay for ‘the bliss of ignorance’. Extending the knowledge on the ostrich effect to the COVID-19 outbreak, it is easy to see how it might have been possible for Italians to ignore the risk of the disease. It may appear, in its most extreme form, in the so called “COVID deniers”, who maintain that COVID-19 does not actually exist, possibly as a way to avoid psychological discomfort [35].

Another cognitive mechanism that might have been activated is the illusion of control; that is, people’s tendency to overestimate their ability to control events, where there is actually little or no control at all [36]. For example, people driving at high speed tend to believe that they have the ability to keep the risk of getting involved in an accident under control. As another example, gamblers in casinos throw the dice harder when they need high numbers, and softer when they need low numbers [37,38]. A further illustration of the illusion of control comes from research on smokers, as follows: only 15% of occasional smokers believe that they will ever become heavy smokers, but 43% of occasional smokers do, in fact, become heavy smokers, within five years [39]. In line with the above findings, during COVID-19, individuals might have erroneously believed that they could keep the risk of contracting the infection under control, and this cognitive distortion might have produced the behavioral response to refuse the recommended public health measures to contain the spreading of the virus.

Two very closely related biases are illusory superiority and the optimism bias (also known as unrealistic optimism). Illusory superiority is the tendency to believe that one has superior qualities and abilities compared to other people [40]. For example, a large majority of people believe their driving skills are above average [41,42]. Optimism bias is the tendency to believe that we are less likely to experience a negative event, compared to others [43]. For example, individuals driving without a seatbelt tend to believe that they are less likely to have an injury, compared to other individuals driving without a seatbelt [44]. According to this illusory superiority, Italians in the coronavirus outbreak could have erroneously believed that their ability to contain and fight the disease was higher than the ability of the Chinese or other countries to do so. According to the optimism bias, Italians might have considered it less likely for the virus to hit Italy, compared to other countries, or for themselves to catch the disease, compared to other people. Our claim is in line with a study conducted in the United States, showing that during the first week of the COVID-19 pandemic, individuals perceived themselves at lower risk of infection than the average person [45].

Another influence on people’s response to COVID-19 might have been the status quo bias, which is the tendency to prefer the current state of things over alternative options [46]. For example, when faced with various decision-making problems, participants tended to remain with the pre-existing baseline, rather than selecting a new, potentially better, option [47]. There is, by now, a large body of research showing that the status quo bias heavily affects human decision-making, across many domains, from ordering the same meals at restaurants, to renewing the same insurance and service providers, rather than risk choosing an unfamiliar, but potentially better, alternative [48]. During COVID-19, the status quo bias might have contributed to people’s resistance to change and their maintaining the same life habits and routines (e.g., shaking hands, going shopping without face coverings, traveling, eating at restaurants with family and friends, celebrating birthdays and weddings with big parties), rather than observing the new social distancing suggestion and stay-at-home order.

A further bias that might explain the behavioral response of Italians during the coronavirus disease pandemic is rationalization, which consists of justifying one’s unacceptable or irrational behavior in a logical way so that it appears rational and right [49]. A typical example is the tendency to explain an irrational purchase (i.e., something unnecessary and very expensive) in a rational way, in order to reduce buyer’s remorse. During the COVID-19 outbreak, individuals who were refusing to observe the restrictions introduced, to help stop the spread of the coronavirus, tried to explain their actions in a logical way, saying, for example, that they had essential tasks to do outside, that they could not postpone their very important trips, that being in company with friends and family was healthier than isolating themselves, and so on.

In addition to the above cognitive mechanisms, the reactance bias, which is our tendency to go in the opposite direction when we perceive our freedom as limited, might have contributed to the low willingness of Italians to adopt the imposed safety measures for the containment of COVID-19. More specifically, psychological reactance is the unpleasant feeling that individuals have when they perceive that their freedom is threatened by someone or something. As a response, they tend to go in the opposite direction, to re-establish their behavioral freedom [50,51]. Brehm and Sensenig [50] discovered psychological reactance in a game that required cooperation among participants. One of these participants was actually a confederate of the researchers and was instructed to pass a note to another participant. In one condition, this note *suggested* a preference among a list of possible courses of actions. In another condition, the note *imposed* a particular course of action. It was observed that about 70% of participants accepted what was suggested, whereas only about 40% of participants accepted what was imposed. During the COVID-19 outbreak, the numerous impositions (i.e., wearing face coverings, social distancing, stay-at-home ordering) coming from authorities, politicians, scientists and public health experts produced a motivation to push back these orders, in an attempt to re-establish one’s own freedom. As a consequence, people not only did not adopt the recommendations about the prevention and control of COVID-19, but suddenly started to crave activities that they were not very interested in before. Interestingly, we observed that certain forbidden activities that were not very exciting for some people before COVID-19 suddenly became highly desirable. For example, people who never had the habit to run, became extremely interested in running, simply because running was one of the forbidden activities. In the same way, people who were never interested in going shopping started had a desire to go shopping. Like in Dostoevsky’s mind game, where his brother could not stop thinking about a white bear simply because he was told not to think of a white bear [52], it is plausible that Italians, placed in front of a series of limitations, started to think more often of (and desire to engage in) those activities that were prohibited [53].

Finally, the exponential growth bias could be the reason for the general under-evaluation of both the risks associated to COVID-19 and the potential benefits from the adoption of preventive behaviors. It seems that people struggle to understand exponential growth, represented by either numbers or graphs [54]. More specifically, Wagenaar and Sagaria [54] presented participants with samples of a hypothetical growth process, over the past 5 years, and asked them to make their intuitive prediction for the next year(s). Participants systematically provided estimates below the numbers that would have fit the correct mathematical model. Individuals, thus, systematically under-estimated exponential growth processes. This bias explains, for example, why people tend to underestimate the long-term benefits of investments or of a retirement plan. In the case of COVID-19, an inability to understand exponential growth translates into an inability to understand how many individuals can be affected by one positive patient, and how many infections could be avoided if one person adheres to COVID-19 preventive behaviors. The under-evaluation of these effects might leave people with a low motivation to engage in preventive behaviors. Our assumption is in line with Banerjee et al.’s work [55], conducted in Germany, U.S.A., France, and Spain, showing that the exponential growth prediction bias, that is, the systematic underestimation of the future number of COVID-19 cases, given the actual data on the disease growth, is a significant predictor of compliance with safety measures, such as wearing masks and practicing social distancing.

## 3. Solutions

In light of the high levels of uncertainty during the outbreak of the COVID-19 pandemic, the activation of heuristics and biases explains very well why many Italians were acting in ways that were not in their own interest or in the interest of their community.

Given the alarming spread of the coronavirus and the growing number of victims that it is still affecting, but also with regards to future pandemics, it appears crucial to help people think more rationally [56]. A first public health intervention to help people behave more logically is to teach them the particular way in which the human mind works, showing with practical examples how people’s thinking and behavior deviates from formal rationality. Raising people’s awareness of the fact that their mind tricks them in many important ways is already a big step towards better decision-making. For example, in medical decision-making, it has been shown that explicit instructions aimed at improving clinicians’ awareness of cognitive biases, significantly reduces diagnostic errors [57]. Policymakers could realize such educational interventions through campaigns, messages, advertisements, videos and appropriate training sessions, specifically designed to improve lay people’s awareness and understanding of the most common cognitive biases. Previous research has shown that even a single training session, presented either as an interactive computer game or an educational video, reduced many biases, in both the short and long-term and in different contexts [58].

A second intervention would be to make use of nudges that lead people towards better decisions [59]. The effectiveness of nudging for improving health behaviors has been shown in prior literature. Even small alterations in the decision-making environment (also known as “choice architecture”) significantly promotes optimal health decisions, such as a higher willingness to participate in a diabetes intervention [60], to get a vaccine [61,62], to use sunscreen [63], or to eat more vegetables [64]. For example, in a self-service cafeteria, it has been observed that simply changing the position of vegetables, so they can be easily reachable, and positioning the more calorie-dense food in a difficult-to-reach location, leads diners to buy healthier meals [64]. Based on these findings, a possible intervention to improve the use of masks and hand washing, during the current pandemic, could be to place free disposable masks and hand sanitizers in grocery stores, malls, offices, and public places, so that it is easy for citizens to adhere to COVID-19 preventive behaviors. Unfortunately, quite the opposite happened in Italy, where the government imposed the use of masks, but masks were not widely available in pharmacies or stores and, when available, they were not sold at a reasonable price [65,66,67,68].

A further intervention could consist of exploiting the very biases we described above, in order to boost compliance with preventive behavior. For example, highlighting examples of many other people wearing masks could activate the bandwagon fallacy and lead to imitations of this desirable behavior. Another example could be to highlight examples of people who got heavily infected after not wearing masks, which could activate the availability heuristic and lead to more people wearing masks.

Another way to help people act more rationally and, thus, to increase their willingness to adhere to COVID-19 preventive behaviors is the use of clear and effective communication, which takes into consideration the limitations of human cognition [69]. In particular, communication should avoid information and choice overload [70,71,72,73]. Communication should be simple, and it should illustrate what people should do and not do, in a way that it is easy to understand for everyone. Giving people too much information and too many choices can lead to the effect of people avoiding making decisions altogether, if they become overwhelmed by the complexity [74,75,76,77,78,79].

Messages should be built taking into account not only our cognitive limitations in processing a large amount of information, but also the fact that many individuals are low in numeracy, which is the ability to comprehend and use basic probability and numerical concepts [80,81]. Research findings showed that numeracy is related to rational decision-making. In particular, individuals low in numeracy are more susceptible to certain cognitive biases and make worse health decisions when these decisions imply the understanding of basic mathematical concepts [82]. Interestingly, Gurmankin et al. [83] observed that individuals low in numeracy trusted verbal risk information more than numeric risk information from physicians.

Given these limitations, as well as the fact that people struggle to understand exponential growth [54,55], any information about risks (such as transmission rate, number of infected people, number of deaths) should be presented in a way that does not require high numerical ability to be understood, nor high predictive exponential growth abilities. As an alternative strategy, research recently conducted in Ireland showed that messages highlighting the risk of COVID-19 transmission to identifiably vulnerable people (such as an elderly person, someone with a previous health condition, or a worker in healthcare) were more effective in promoting social distancing than messages highlighting the exponential transmission rate of COVID-19 [84]. The cognitive bias activated in this case refers to the higher willingness of individuals to help specific identifiable victims, rather than victims that are described statistically [85].

When statistics are communicated, it is essential that their meaning for both the individual and the population are properly explained. Importantly, information should always be represented in terms of frequencies (5 out of 10 people are affected), rather than probabilities (50% of people are affected). An abundance of research has shown that frequency formats dramatically enhance people’s performance on probabilistic tasks [86,87]. There are two main reasons why frequencies are easier to process than probabilities, one evolutionary and one computational [88]. From an evolutionary perspective, natural frequencies have always been the way in which humans have experienced numerical information in the visible and tangible world, whereas probabilities are an abstract concept. Probabilistic reasoning is, therefore, a relatively recent achievement in human history. From a computational point of view, frequencies involve operations with whole numbers, rather than with fractions, which makes them simpler and easier to process.

Moreover, communication should take into account individual differences in decision-making. For example, decision-makers can be distinguished as maximizers or satisficers; the former spend substantial time and effort analyzing all information available in order to make the best choice; the latter instead evaluate a smaller amount of information, spending less time and effort, to find a good enough option [89,90,91]. Interestingly, it seems that maximizers and satisficers differ in the way they weigh desirability and feasibility in their decision-making [92]. Desirability is the benefit derived from a given option, whereas feasibility refers to the difficulty of obtaining that option. Research findings show that maximizers focus more on desirability than feasibility aspects [93] and, thus, they tend to choose options that are highly desirable, even if difficult to reach (e.g., a beautiful vacation destination, even though it is very far). On the contrary, satisficers consider feasibility as more important than desirability and, thus, prefer options that are more feasible, even though less desirable (e.g., a closer vacation destination that it is good enough). Based on these findings, public health policymakers should generate messages, campaigns and advertisements that speak to both maximizers and satisficers, by highlighting both the desirability of adopting the suggested preventive behaviors (e.g., how many lives can be saved) as well as the feasibility of adhering to such behaviors (e.g., how easy it is to adhere to preventive behaviors).

Finally, communication should be transparent and avoid misinformation, for at least two important reasons. Transparency and truthfulness are key elements in establishing trust among the interlocutors. As a direct consequence, trust in the government would increase individuals’ willingness to cooperate with official guidelines, to fight the COVID-19 pandemic. In addition, though, a transparent and truthful communication reduces uncertainty and, with that, the need for people to use heuristics, rather than a more rational decision process.

Unfortunately, misinformation is now a big concern of public interest. It is spread in society, either inadvertently or purposely, by media, politicians, governments, rumors, and even corporations. The most dangerous aspect of misinformation is that, once disseminated, it is very difficult to correct [94]. Research showed that interventions aimed at rectifying misinformation are mostly ineffective. For example, in 1998, in the United Kingdom, the false belief was spread about a link between childhood vaccines and autism. Even after years of corrective campaigns, showing the lack of evidence of such a link, many continued to reject vaccines for their children [95]. Because erroneous beliefs persist even after expensive corrective actions, avoiding misinformation is very important when messages about the pandemic are given to the public. For example, the incorrect information provided by public health experts and scientists at the beginning of the epidemic, that COVID-19 was nothing more than a common flu, persisted for months, even as the number of deaths and infected people were clearly showing the invalidity of such a claim. In conclusion, as recently stated in the Lancet [96], “There may be no way to prevent a COVID-19 pandemic in this globalized time, but verified information is the most effective prevention against the disease of panic”.

The present manuscript focused on Italians’ response to the COVID-19 emergency. However, similar considerations could be extended to irrational decisions made in any other country, as cognitive biases are notoriously culture independent [97].

## Data Availability

Not applicable.

## References

[1] Libero Quotidiano Coronavirus, Vittorio Sgarbi Contro I Virology: “Cosa Dimostra il Caso di Porro e Zingaretti”. Libero Quotidiano. https://www.liberoquotidiano.it/news/personaggi/20953716/coronavirus_vittorio_sgarbi_contro_virologi_caso_porro_zingaretti_dimostra_non_e_letale.html.

[2] Marrone C. Coronavirus, la Direttrice del Laboratorio Sacco: <<State Calmi, una Follia che Farà Male>>. La Risposta di Burioni. Corriere della Sera. https://www.corriere.it/salute/malattie_infettive/20_febbraio_23/coronavirus-direttrice-laboratorio-sacco-non-esagerate-state-calmi-follia-che-fara-male-72442dca-562d-11ea-b447-d9646dbdb12a.shtml.

[3] Open La Professoressa del Sacco: <<Ora in Tanti mi Danno Ragione sul Coronavirus, ne Farò un Ciondolo>>. Open. https://www.open.online/2020/02/26/la-dottoressa-del-sacco-ora-in-tanti-mi-danno-ragione-fra-una-settimana-non-parleremo-piu-di-coronavirus-ne-faro-un-ciondolo/.

[4] Sardiniapost Differenze tra Coronavirus e Influenza: Ecco Tutto Quello che Bisogna Sapere. Sardiniapost. https://www.sardiniapost.it/cronaca/differenze-tra-coronavirus-e-influenza-ecco-tutto-quello-che-bisogna-sapere/.

[5] Verdi C. Quando Zingaretti Diceva: “Coronavirus? L’allarmismo è Infondato”. Il Giornale. https://www.ilgiornale.it/news/politica/quando-zingaretti-diceva-coronavirus-lallarmismo-infondato-1838116.html.

[6] Bartoloni M. Coronavirus: Nell’80–90% Dei Casi è Come l’Influenza, per Gli Altri Rischio Polmonite. Il Sole24ore. https://www.ilsole24ore.com/art/coronavirus-nell-80-90percento-casi-e-come-l-influenza-gli-altri-rischio-polmonite-ACQ4pMLB.

[7] La Repubblica Coronavirus, Zingaretti Aperitivo Pubblico a Milano: “Niente Panico, Isolare i Focolai. Il Governissimo? Non c’è la Crisi”. La Repubblica. https://www.repubblica.it/politica/2020/02/27/news/coronavirus_zingaretti_contro_il_panico-249718891/.

[8] Nussbaumer-Streit B., Mayr V., Dobrescu A.I., Chapman A., Persad W., Klerings I., Wagner G., Siebert U., Christof C., Zachariah C. (2020). Quarantine alone or in combination with other public health measures to control COVID-19: A rapid review. Crochrane Database Syst. Rev..

[9] Gagliardi A. Da Inutili a Obbligatorie Anche All’aperto, il Cambio di Rotta Sulle Mascherine. Il Sole24ore. https://www.ilsole24ore.com/art/da-inutili-obbligatorie-anche-all-aperto-cambio-rotta-mascherine-ADrrTSu.

[10] Giuliani F. Coronavirus, il Viceministro Sileri: “Le Mascherine Non Servono”. Il Giornale. https://www.ilgiornale.it/news/mondo/coronavirus-sileri-usare-mascherine-stupidaggine-enorme-1819425.html.

[11] Salvadorini R. Coronavirus, le Mascherine sono Utili o Solo un Ansiolitico? Il Punto non è l’Efficacia, ma I Rischi. Il Fatto Quotidiano. https://www.ilfattoquotidiano.it/2020/04/16/coronavirus-le-mascherine-sono-utili-o-solo-un-ansiolitico-il-punto-non-e-lefficacia-ma-i-rischi/5770981/.

[12] Tversky A., Kahneman D. (1974). Judgment under uncertainty: Heuristics and biases. Science.

[13] Di Giacomo V. Coronavirus, da Milano a Napoli gli Irresponsabili della Movida: Solita Folla nei Locali. Il Mattino. https://www.ilmattino.it/napoli/cronaca/coronavirus_a_napoli_e_in_italia-5099282.html.

[14] Di Raimondo R. Bonaccini: “Negazionisti? Cialtroni Irresponsabili”. La Repubblica. https://bologna.repubblica.it/cronaca/2020/09/06/news/coronavirus_124_casi_in_piu_in_emilia-romagna_salgono_ancora_i_ricoveri-266404293/.

[15] Riccio I. De Luca: “Aveva il Coronavirus ed è Andato a Ballare: Comportamenti Irresponsabili”. Il Giornale. https://www.ilgiornale.it/news/napoli/de-luca-aveva-coronavirus-ed-andato-ballare-comportamenti-1837287.html.

[16] Sir Coronavirus COVID-19: Coldiretti/Ixè, il 27% degli Italiani rifiuta la mascherina o la indossa raramente. Sir. https://www.coldiretti.it/economia/mascherina-1-italiano-su-4-la-rifiuta.

[17] Lichtenstein S., Slovic P., Fishhoff B., Layman M., Combs B. (1978). Judged frequency of Lethal Events. J. Exp. Psychol. Hum. Learn. Mem..

[18] Rothman A.J., Klein W.M., Weinstein N.D. (1996). Absolute and Relative Biases in Estimations of Personal Risk. J. Appl. Soc. Psychol..

[19] Tewksbury D., Scheufele D.A., Oliver M.B., Raney A.A., Bryant J. (2020). News Framing Theory and Research. Media Effects: Advances in Theory and Research.

[20] Trussler M., Soroka S. (2014). Consumer demand for cynical and negative news frames. Int. J. Press Politics.

[21] Giangrande A. (2020). L’Italia Allo Specchio. Il DNA Degli Italiani Anno 2020. Gli Statisti Prima Parte.

[22] Demos Gli Italiani e lo Stato. Rapporto 2019. Demos. http://www.demos.it/rapporto.php.

[23] Giangrande A. (2020). Malagiustiziopoli. Seconda Parte: Malagiustizia. Disfunzioni del Sistema.

[24] Asch S.E., Guetzkow H. (1951). Effects of group pressure on the modification and distortion of judgments. Groups, Leadership and Men.

[25] Farjam M. (2020). The bandwagon effect in an online voting experiment with real political organizations. Int. J. Public Opin. Res..

[26] Maldonato M., Dell’Orco S. (2011). How to make decisions in an uncertain world: Heuristics, biases, and risk perception. World Futures.

[27] Steinberg L., Monahan K.C. (2007). Age differences in resistance to peer influence. Dev. Psychol..

[28] Calpista R. L’insostenibile Leggerezza Dell’essere (Irresponsabili). La Gazzetta del Mezzogiorno. https://www.lagazzettadelmezzogiorno.it/news/analisi/1224769/linsostenibile-leggerezza-dellessere-irresponsabili.html.

[29] Il Dolomiti Coronavirus, Organizzano una Partita a Tennis in Cimirlo. Due Giovani Denunciati. Il Dolomiti. https://www.ildolomiti.it/cronaca/2020/coronavirus-organizzano-una-partita-a-tennis-in-cimirlo-due-giovani-denunciati.

[30] Wason P.C., Foss B.M. (1966). Reasoning. New Horizons in Psychology.

[31] Cardaci M., Misuraca R. (2005). Rethinking of the Heuristic-Analytic Dual Process Theory: A comment on Wada and Nittono (2004) and the reasoning process in the Wason Selection Task. Percept. Mot. Ski..

[32] Grosset N., Barrouillet P., Misuraca R. (2004). Development of conditional reasoning and Wason’s selection task (Développement du raisonnement conditionnel et tâche de sélection de Wason). Année Psychol..

[33] Karlsson N., Loewenstein G., Seppi D. (2009). The ostrich effect: Selective attention to information. J. Risk Uncertain..

[34] Galai D., Sade O. (2006). The “Ostrich Effect” and the Relationship between the Liquidity and the Yields of Financial Assets. J. Bus..

[35] Kahn-Harris K. (2018). Denial: The Unspeakable Truth.

[36] Langer E.J. (1975). The illusion of control. J. Personal. Soc. Psychol..

[37] Henslin J.M. (1967). Craps and Magic. Am. J. Sociol..

[38] Plous S. (1993). McGraw-Hill Series in Social Psychology. The Psychology of Judgment and Decision Making.

[39] McKenna F.P., Warburton D.M., Winwood M. (1993). Exploring the limits of optimism: The case of smokers’ decision making. Br. J. Psychol..

[40] Buunk B.P., Van Yperen N.W. (1991). Referential comparisons, relational comparisons, and exchange orientation: Their relation to marital satisfaction. Personal. Soc. Psychol. Bull..

[41] Svenson O. (1981). Are we all less risky and more skillful than our fellow drivers?. Acta Psychol..

[42] McCormick I.A., Walkey F.H., Green D.E. (1986). Comparative perceptions of driver ability: A confirmation and expansion. Accid. Anal. Prev..

[43] Weinstein N.D. (1980). Unrealistic optimism about future life events. J. Personal. Soc. Psychol..

[44] Svenson O., Fishhoff B., McGregor D. (1985). Perceived driving safety and seatbelt usage. Accid. Anal. Prev..

[45] Wise T., Zbozinek T.D., Michelini G., Hagan C.C., Mobbs D. (2020). Changes in risk perception and self-reported protective behavior during the first week of the COVID-19 pandemic in the United States. R. Soc. Open Sci..

[46] Kahneman D., Knetsch J.L., Thaler R.H. (1991). Anomalies: The Endowment Effect, Loss Aversion, and Status Quo Bias. J. Econ. Perspect..

[47] Samuelson W., Zeckhauser R. (1988). Status quo bias in decision making. J. Risk Uncertain..

[48] Ortoleva P. (2010). Status quo bias, multiple priors and uncertainty aversion. Games Econ. Behav..

[49] Brehm J.W. (1956). Post-decisional changes in the desirability of alternatives. J. Abnorm. Soc. Psychol..

[50] Brehm J.W., Sensenig J. (1966). Social influence as a function of attempted and implied usurpation of choice. J. Personal. Soc. Psychol..

[51] Steindl C., Jonas E., Sittenthaler S., Traut-Mattausch E., Greenberg J. (2015). Understanding psychological reactance: New developments and findings. Z. Für Psychol..

[52] Wegner D.M., Schneider D.J., Carter S.R., White T.L. (1987). Paradoxical effects of thought suppression. J. Personal. Soc. Psychol..

[53] Misuraca R., Ceresia F. (2020). Psychological Reactance as an explanation of Italians’ resistance to observe the safety measures during COVID-19 outbreak. Rev. De Cienc. Soc..

[54] Wagenaar W.A., Sagaria S.D. (1975). Misperception of exponential growth. Percept. Psychophys..

[55] Banerjee R., Bhattacharya J., Majumdar P. (2021). Exponential growth prediction bias and compliance with safety measures related to COVID-19. Soc. Sci. Med..

[56] Miceli S., Maniscalco S., Matranga D. (2019). Social networks and social activities promote cognitive functioning in both concurrent and prospective time: Evidence from the SHARE survey. Eur. J. Ageing.

[57] Royce C.S., Haynes M.M., Schwartzstein R.M. (2019). Teaching critical thinking: A case for instruction in cognitive biases to reduce diagnostic errors and improve patient safety. Acad. Med..

[58] Morewedge C.K., Yoon H., Scopelliti I., Symborski C.W., Korris J.H., Kassam K.S. (2015). Debiasing Decisions: Improved Decision Making With a Single Training Intervention. Policy Insights Behav. Brain Sci..

[59] Thaler R.H., Sunstein C.R. (2008). Nudge: Improving Decisions about Health, Wealth and Happiness.

[60] Aysola J., Tahirovic E., Troxel A.B., Asch D.A., Gangemi K., Hodlofski A.T., Zhu J., Volpp K. (2018). A randomized controlled trial of opt-in versus opt-out enrollment into a diabetes behavioral intervention. Am. J. Health Promot..

[61] Li M., Chapman G.B. (2009). 100% of anything looks good: The appeal of one hundred percent. Psychon. Bull. Rev..

[62] Chapman G.B., Li M., Colby H., Yoon H. (2010). Opting in vs. opting out of influenza vaccination. JAMA.

[63] Li M., Chapman G.B. (2013). Nudge to Health: Harnessing Decision Research to Promote Health Behavior. Soc. Personal. Psychol. Compass.

[64] Rozin P., Scott S., Dingley M., Urbanek J.K., Jian H., Kaltenback M. (2011). Nudge to nobesity I: Minor changes in accessibility decrease food intake. Judgm. Decis. Mak..

[65] Basso L. Mascherine, Prezzi Ancora Alti Fino al Triplo del Tetto Stabilito dal Commissario. L’Adige. https://www.ladige.it/news/cronaca/2020/04/28/mascherine-prezzi-farmacia-ancora-alti.

[66] Cosimi S. Coronavirus, Prezzi Alle Stelle per Gel e Mascherine: Amazon Bacchetta Gli Speculatori in Italia e All’estero. La Repubblica. https://www.repubblica.it/tecnologia/prodotti/2020/02/25/news/prezzi_alle_stelle_amazon_bacchetta_gli_speculatori_in_italia_e_all_estero-249620527/.

[67] Nuti V. Introvabili, Care, Non Certificate: Il Gran Caos delle Mascherine Nella Fase 2. Il Sole24ore. https://www.ilsole24ore.com/art/mascherine-pasticcio-all-italiana-prezzi-popolari-ma-introvabili-certificate-o-anche-fai-te-AD5LSLP.

[68] Rifday Altroconsumo, Nuova Rilevazione sul Mercato delle Mascherine: Si Trovano, ma Sono Ancora Care. Rifday. https://www.rifday.it/2020/04/23/altroconsumo-nuova-rilevazione-sul-mercato-delle-mascherine-si-trovano-ma-sono-ancora-care/.

[69] Simon H.A. (1955). A behavioral model of rational choice. Q. J. Econ..

[70] Fasolo B., Carmeci F.A., Misuraca R. (2009). The effect of choice complexity on perception of time spent choosing: When choice takes longer but feels shorter. Spec. Issue Assortment Struct. Choice Psychol. Mark..

[71] Misuraca R., Ceresia F., Nixon A.E., Scaffidi Abbate C. (2021). When is more really more? The effect of brands on choice overload in adolescents. J. Consum. Mark..

[72] Misuraca R., Faraci P., Ruthruff E., Ceresia F. (2021). Are maximizers more normative decision-makers? An experimental investigation of maximizers’ susceptibility to cognitive biases. Personal. Individ. Differ..

[73] Reutskaja E., Iyengar S.S., Fasolo B., Misuraca R., Viale R. (2021). Cognitive and affective consequences of information and choice overload. Handbook of Bounded Rationality.

[74] Misuraca R. (2013). Do too many choices have negative consequences? An empirical review. (Troppa scelta ha veramente conseguenze negative? Una rassegna di studi empirici). G. Ital. Di Psicol..

[75] Misuraca R., Ceresia F., Teuscher U., Faraci P. (2019). The role of the brand on choice overload. Mind Soc. Cogn. Stud. Econ. Soc. Sci..

[76] Misuraca R., Faraci P. (2021). L’effetto del sovraccarico di scelta: Un’indagine su bambini, adolescenti, adulti e anziani. Ric. Di Psicol..

[77] Misuraca R., Miceli S., Teuscher U. (2017). Three effective ways to nurture our brain: Physical activity, healthy nutrition, and music. A review. Eur. Psychol..

[78] Misuraca R., Teuscher U., Faraci P. (2016). Is more choice always worse? Age differences in the overchoice effect. J. Cogn. Psychol..

[79] Anderson B.F., Misuraca R. (2017). Perceptual Commensuration in Decision Tables. Q. J. Exp. Psychol..

[80] Paulos J.A. (1988). Innumeracy: Mathematical Illiteracy and its Consequences.

[81] Misuraca R., Teuscher U., Carmeci F.A. (2016). Who are maximizers? Future oriented and highly numerate individuals. Int. J. Psychol..

[82] Hamm R.M., Bard D.E., Scheid D.C. Influence of numeracy upon patient’s prostate cancer screening outcome probability judgments. Proceedings of the Annual Meeting of the Society for Judgment and Decision Making.

[83] Gurmankin A.D., Baron J., Armstrong K. (2004). The effect of numerical statements of risk on trust and comfort with hypothetical physician risk communication. Med. Decis. Mak..

[84] Lunn P.D., Timmons S., Barjaková M., Belton C.A., Julienne H., Lavin C. (2020). Motivating Social Distancing during the Covid-19 Pandemic: An Online Experiment. Soc. Sci. Med..

[85] Jenni K.E., Loewenstein G. (1997). Explaining the “Identifiable Victim Effect”. J. Risk Uncertain..

[86] Gigerenzer G., Hoffrage U. (1995). How to improve Bayesian reasoning without instruction: Frequency formats. Psychol. Rev..

[87] Misuraca R., Cardaci M. (2005). Frequency format facilitates reasoning in simple numerical tasks. Psychol. Rep..

[88] Misuraca R., Carmeci F.A., Pravettoni G., Cardaci M. (2009). Facilitating effect of natural frequencies: Size does not matter. Percept. Mot. Ski..

[89] Misuraca R., Teuscher U. (2013). Time flies when you maximize. Maximizers and satisficers perceive time differently when making decisions. Acta Psychol..

[90] Misuraca R., Faraci P., Gangemi A., Carmeci F.A., Miceli S. (2015). The Decision Making Tendency Inventory: A new measure to assess maximizing, satisficing, and minimizing. Personal. Individ. Differ..

[91] Misuraca R., Reutskaja E., Fasolo B., Iyengar S.S., Viale R. (2021). How much choice is “good enough”? Moderators of information and choice overload. Handbook of Bounded Rationality.

[92] Misuraca R., Fasolo B. (2018). Maximizing versus satisficing in the digital age: Disjoint scales and the case for “construct consensus”. Personal. Individ. Differ..

[93] Hsieh M.H., Yalch R., Love E., Diehl K., Yoon C. (2015). The influence of a maximizing versus satisficing orientation on the evaluation of desirability and feasibility attributes. NA—Advances in Consumer Research.

[94] Lewandowsky S., Ecker U.K.H., Seifert C.M., Schwarz N., Cook J. (2012). Misinformation and its correction: Continued influence and successful debiasing. Psychol. Sci. Public Interest.

[95] Hargreaves I., Lewis J., Speers T. (2003). Towards a Better Map: Science, the Public and the Media.

[96] The Lancet (2008). COVID-19: Fighting panic with information. Lancet.

[97] Kahneman D. (2011). Thinking, Fast and Slow.

